# CD36 Inhibits Triple‐Negative Breast Cancer Progression by Transcriptionally Upregulating Caveolin‐1 and Promoting Lipid‐Reactive Oxygen Species‐Related Ferroptosis

**DOI:** 10.1002/mco2.70493

**Published:** 2025-11-18

**Authors:** Xiujuan Wu, Yan Wang, Zaihui Peng, Tingting Zhao, Xuanni Tan, Wenting Yan, Yuqin Zhou, Jie Xia, Xiaowei Qi, Yi Zhang

**Affiliations:** ^1^ Department of Breast and Thyroid Surgery/Key Laboratory of Chongqing Health Commission for Minimally Invasive and Precise Diagnosis and Treatment of Breast Cancer Southwest Hospital, Army Medical University Chongqing China; ^2^ Key Laboratory of Molecular Biology For Diseases (Ministry of Education), Institute for Viral Hepatitis, Department of Infectious Diseases, The Second Affiliated Hospital, Chongqing Medical University Chongqing China; ^3^ Western(Chongqing) Collaborative Innovation Center for Intelligent Diagnostics and Digital Medicine Chongqing National Biomedicine Industry Park Chongqing China

**Keywords:** caveolin‐1, CD36, ferroptosis, lipid‐reactive oxygen, triple‐negative breast cancer

## Abstract

Triple‐negative breast cancer (TNBC) is an aggressive subtype with limited therapeutic options and poor prognosis. Cluster of differentiation 36 (CD36), a fatty acid transporter, plays controversial roles in tumor progression. Here, we report a tumor‐suppressive function of CD36 in TNBC. Analysis of The Cancer Genome Atlas and Gene Expression Omnibus databases, along with validation in clinical samples, revealed that CD36 expression was significantly downregulated in TNBC tissues, and its low expression correlated with advanced disease stage and poorer patient prognosis. Functional assays demonstrated that CD36 knockout promoted, whereas its overexpression inhibited, the proliferation, migration, and invasion of TNBC cells. Integrated transcriptomic and proteomic analyses linked CD36 to ferroptosis, an iron‐dependent form of regulated cell death. Mechanistically, CD36 enhanced the transcriptional activity of peroxisome proliferator‐activated receptor gamma (PPARγ), which in turn upregulated the expression of caveolin‐1 (CAV1). This CD36/PPARγ/CAV1 axis increased intracellular lipid peroxidation, thereby promoting ferroptosis. In vivo, a CD36 agonist suppressed, while a ferroptosis activator inhibited the metastasis of CD36‐knockdown TNBC cells. Our findings identify CD36 as a novel tumor suppressor in TNBC that acts by promoting ferroptosis, highlighting its potential as both a prognostic biomarker and a therapeutic target.

## Graphical abstract

CD36 is identified as a key suppressor in triple‐negative breast cancer (TNBC). It inhibits tumor metastasis by promoting ferroptosis via the CD36–CAV1–lipid peroxidation axis. Multiomics and metastasis models confirm that CD36 activation exerts therapeutic effects, highlighting its promise as a novel gene therapy target for TNBC.

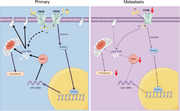

## 1 Introduction

As of 2024, breast cancer accounts for 32% of all cancers among women in the United States, up from 31% in 2023 [1]. Among these cases, triple‐negative breast cancer (TNBC) constitutes approximately 20–25% of all breast cancers and is associated with a poor prognosis [2, 3, 4, 5]. TNBC patients do not benefit from molecular targeted therapies, such as endocrine therapy or trastuzumab, due to the absence of suitable targets for these drugs. Given the poor prognosis associated with this subtype of breast cancer, identifying targets that regulate the growth and metastasis of TNBC cells and developing effective interventions are of significant importance for its treatment.

Cluster of differentiation 36 (CD36) is a membrane glycoprotein composed of a single polypeptide chain of 472 amino acids, with a molecular weight of 88 kDa. It is not universally expressed but is present in various mammalian cell types [6]. CD36 is involved in multiple cellular functions, including lipid metabolism, inflammatory responses, and the clearance of apoptotic cells [7]. Its expression has been specifically correlated with tumor metastasis. In most cancer studies, CD36 primarily functions as a fatty acid (FA) transporter, mediating the uptake of extracellular FAs to support the metabolic needs of cancer cell proliferation and metastasis [8, 9]. Research by Pascual et al. [10] identified CD36 as a common marker of metastatic cells, currently the first marker specific to metastasis. Additionally, studies have shown that CD36 plays a significant role in tumor angiogenesis, epithelial–mesenchymal transition, and other processes. The role of CD36 in tumors has garnered significant attention; however, its function in cancer remains controversial. Even within the same cancer type, CD36 can act as either an oncogene or a tumor suppressor. For example, in glioblastoma, CD36 overexpression in cancer stem cells promotes cancer progression [11], while endothelial CD36 expression exerts antiangiogenic and proapoptotic effects [12, 13]. Some studies suggest that in pancreatic tumors, CD36 promotes metastasis by regulating immune cell invasion, microvesicle extravasation, and the infiltration of specific tissue macrophages [14]. Conversely, other studies have found that CD36 expression in pancreatic cancer cells and tissues is significantly lower than in corresponding normal tissues, with low CD36 expression predicting larger tumor size and poorer survival prognosis. This suggests that CD36 may serve as a predictor of clinical pathological features and prognosis in pancreatic cancer [15]. These findings indicate that CD36 has unique cell type‐specific, environment‐specific, and function‐specific roles, even within the same type of cancer. In colorectal tumors, CD36 has been found to inhibit tumorigenesis by ubiquitinating glypican‐4 (GPC4), thereby suppressing β‐catenin/c‐myc‐mediated glycolysis [16]. In breast cancer, some studies have indicated that high CD36 expression enhances the proliferation and migration of ER‐positive breast cancer cells while weakening the inhibitory effect of tamoxifen on ER‐positive cell growth [17], suggesting that CD36 plays a role in mediating resistance to ER‐targeted therapies [18]. However, other well‐documented reports have indicated that epithelial, endothelial, or stromal CD36 expression is negatively correlated with breast cancer proliferation and invasiveness [19, 20, 21]. The characteristics and mechanisms of CD36 in the development of TNBC remain unclear.

Dixon et al. [22] discovered that the accumulation or overload of intracellular iron ions, induced by small molecules, leads to lipid peroxidation and membrane structure collapse, resulting in oxidative cell death. They named this type of programmed cell death, which is dependent on iron and lipid peroxides, as ferroptosis [22]. Ferroptosis is a critical cell death mechanism for inhibiting tumor growth. Unlike apoptosis, it is not inhibited by apoptosis inhibitors and is mainly characterized by a significant increase in cytoplasmic iron and lipid peroxidation levels, a reduction in mitochondrial volume, and an increase in double membrane thickness. Ferroptosis levels can be evaluated by detecting lipid peroxidation or by measuring iron abundance and the activity of glutathione peroxidase 4 (GPX4) [23, 24, 25]. In recent years, ferroptosis has attracted considerable attention in oncology research. Regulating cell ferroptosis to intervene in cancer progression has become a new focus in cancer treatment. Compared with normal cells, cancer cells exhibit more active metabolism, mitochondrial dysfunction, and higher reactive oxygen species (ROS) accumulation, making them more sensitive to ferroptosis. To prevent ROS‐induced damage, cancer cells activate stress responses and antioxidant mechanisms to clear excess ROS and reduce ferroptosis levels. Malignant tumor cells, such as those in liver, gastric, lung, pancreatic, colon, and ovarian cancers and melanomas, have been confirmed to be sensitive to ferroptosis, and the use of ferroptosis inducers can effectively inhibit tumor cell growth [26, 27, 28, 29, 30, 31, 32, 33]. Therefore, inducing ferroptosis in cancer cells is considered one of the crucial strategies for inhibiting tumor growth and has significant implications for cancer treatment [34]. In breast cancer research, the latest study by Zhimin et al. found that TNBC exhibits heterogeneous phenotypes in ferroptosis‐related metabolites and metabolic pathways [35]. The luminal androgen receptor subtype of TNBC is characterized by upregulation of oxidized phosphatidylethanolamine and glutathione metabolism, particularly GPX4, suggesting that GPX4 inhibitors can be used to induce ferroptosis. GPX4 inhibition not only induces tumor ferroptosis, but also enhances antitumor immunity [35]. Although it has been reported that ferroptosis levels can affect TNBC progression, the potential regulation by CD36 and the specific regulatory mechanisms have not been discussed.

In this study, we report the reduced expression and tumor‐suppressive role of CD36 in TNBC development. We hypothesized that “ferroptosis in TNBC is closely related to the proliferation and metastasis of TNBC, and that the process of ferroptosis is regulated by CD36 expression.” Additionally, our research provided new mechanistic insights into CD36 regulation of TNBC development. Specifically, CD36 regulated the expression level and protein activity of caveolin‐1 (CAV1) through peroxisome proliferator‐activated receptor gamma (PPARγ), thereby increasing intracellular lipid peroxidation levels, promoting ferroptosis in TNBC cells, and inhibiting the high proliferative growth activity of TNBC.

## 2 Results

### 2.1 Expression and Prognosis of CD36 in Breast Cancer Patients

**FIGURE 1:**
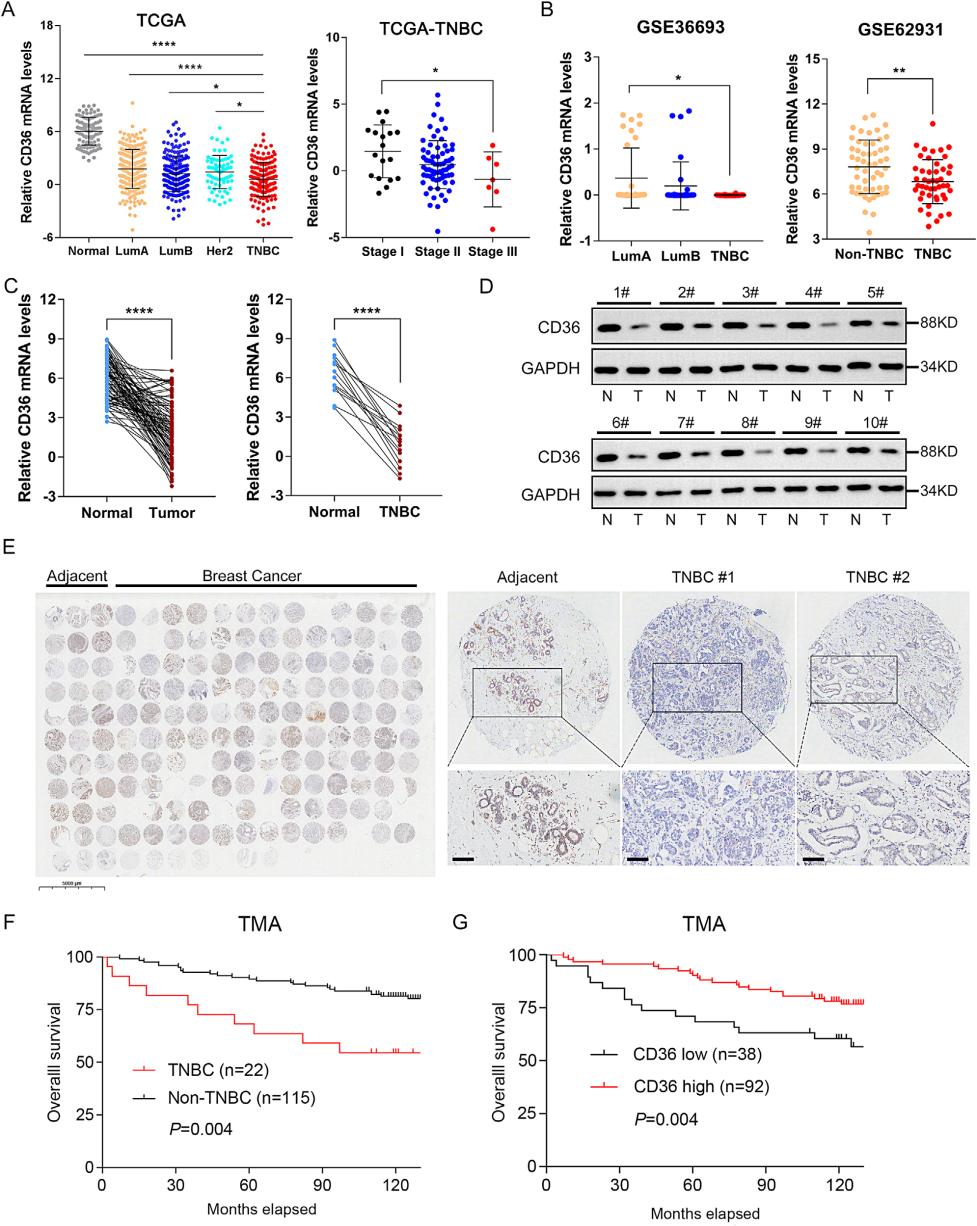
CD36 is downregulated in breast cancer and correlates with poor prognosis. (A) Gene chip data were obtained and compared from TCGA cohorts. (B) Gene chip data were obtained and compared from 2 GEO datasets. Results are shown as mean ± SEM, **p < 0.01, ***p < 0.001, based on Student's *t*‐test. (C) qRT‐PCR analysis of mRNA expression in paired breast cancer tissues. Results were shown as mean ± SEM, ***p < 0.001, based on paired Student's *t*‐test. (D) Western blots of CD36 protein in 10 pairs of TNBC tissues, GAPDH was loaded as a control. (E) Breast cancer tissue microarray (*n* = 170). Immunohistochemistry of CD36 in TNBC tissues and adjacent tissues, scale bar, 50 µm (10×). (F and G) Kaplan–Meier survival curves of tissue microarray analysis.

Recent advances in high‐throughput sequencing have facilitated the analysis of large‐scale biological data, available in databases such as The Cancer Genome Atlas (TCGA) and the Gene Expression Omnibus (GEO). To explore the role of CD36 in breast cancer, we initially analyzed gene expression data from breast cancer patients using the TCGA and GEO databases. A comprehensive analysis of CD36 expression across various tumor types in the TCGA database revealed that CD36 was generally downregulated in most tumors, including bladder cancer, lung adenocarcinoma, and cholangiocarcinoma, while it was upregulated in a few tumors, such as glioblastoma and kidney renal clear cell carcinoma (Figure S1A). Specifically, we observed that CD36 expression was significantly lower in breast cancer tissues compared with normal tissues, with the lowest expression levels found in TNBC subtypes. Moreover, CD36 expression decreased progressively with advancing stages of TNBC (Figure 1A). To validate these findings, we analyzed two GEO datasets, GSE36693 and GSE62931, confirming that CD36 expression was indeed lower in TNBC compared with other breast cancer subtypes (Figure 1B). Further validation at the mRNA and protein levels was achieved through reverse transcription‐quantitative polymerase chain reaction (RT‐qPCR) and western blotting analyses of TNBC tissue samples. These experiments demonstrated that CD36 mRNA and protein levels were lower in TNBC tissues than in adjacent noncancerous tissues (Figure 1C,D). Immunohistochemistry (IHC) analysis of TNBC tissue microarrays corroborated these results, showing reduced CD36 protein expression in TNBC (Figure 1E). Using Kaplan–Meier plotter analysis, we examined the correlation between CD36 expression and breast cancer prognosis. While overall CD36 expression levels did not show a significant correlation with overall survival (OS), relapse‐free survival, and distant metastasis‐free survival in breast cancer patients (Figure S1B), a positive correlation was observed in TNBC subtypes (Figure S1C–F). Finally, analysis of TNBC tissue microarrays revealed that reduced CD36 expression correlated with poorer prognosis in TNBC patients (Figure 1F,G). These findings suggested that CD36 expression was significantly associated with prognosis in TNBC patients, indicating its potential role as a tumor suppressor in this breast cancer subtype.

### 2.2 CD36 Plays Anticarcinogenic Roles in TNBC

**FIGURE 2:**
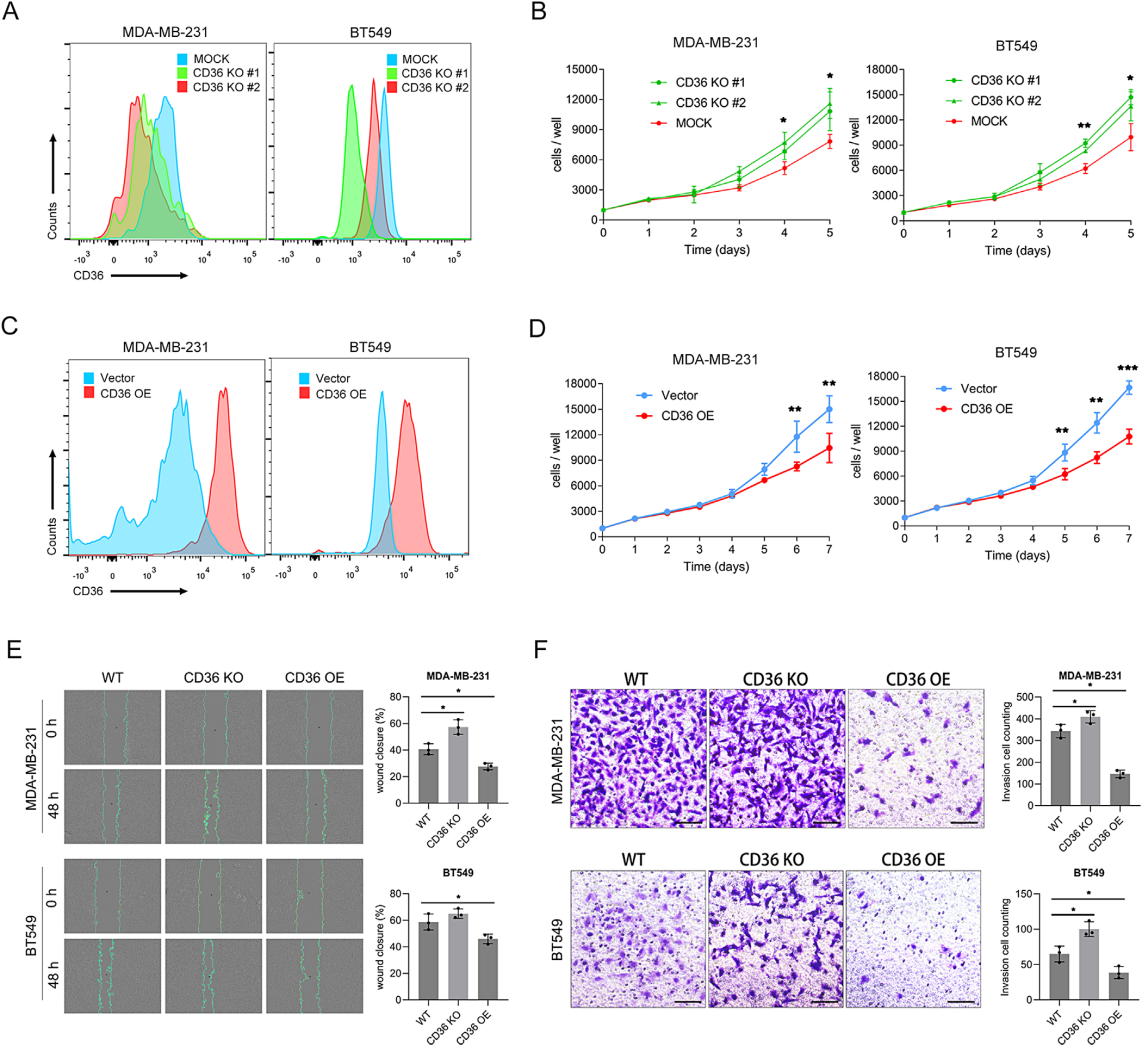
CD36 suppresses TNBC cell proliferation, migration, and invasion. (A) Flow cytometry analysis to identify the effect of CD36 knockout in MDA‐MB‐231 and BT549 cells. (B) Using cell counting methods to evaluate the growth rate of MDA‐MB‐231 and BT549 cells after CD36 knockout. (C) Flow cytometry analysis to identify the effect of CD36 overexpression in MDA‐MB‐231 and BT549 cells. (D) Using cell counting methods to evaluate the growth rate of MDA‐MB‐231 and BT549 cells after CD36 overexpression. (E) Scratch assay of MDA‐MB‐231 cells and BT549 cells with altered CD36 expression. (F) Invasion assay of MDA‐MB‐231 and BT549 cells with altered CD36 expression. Each experiment was performed at least three times, and the results are presented as mean ± standard deviation. Data were analyzed using Student's *t*‐test and one‐way ANOVA (*p < 0.05, **p < 0.01, ***p < 0.001, ****p < 0.0001).

Given previous results, we further investigated the functional role of CD36 in TNBC cells. We first assessed endogenous CD36 protein levels in different breast cancer cell lines. Western blotting analysis confirmed that CD36 expression showed lowest levels in TNBC cell (MDA‐MB‐231, HCC38, and BT549) compared with Luminal A/B (MCF‐7, ZR‐75‐1, T47D) or Her2‐positive (SK‐BR‐3) subtype cells (Figure S2A). We then performed CD36 alteration experiments in MDA‐MB‐231 and BT549 cells, utilizing flow cytometry to assess the effects of these manipulations (Figure 2A,C). Our findings indicated that knockout of CD36 led to a significant increase in the growth rate of both MDA‐MB‐231 and BT549 cells, while overexpression of CD36 significantly decreased cell growth (Figure 2B,D). The impact of CD36 on cell migration was evaluated through scratch assays, revealing that CD36 knockout enhanced migration ability, whereas overexpression reduced it (Figure 2E). Additionally, transwell assays were used to assess the effects on cell invasion. We found that CD36 knockout significantly enhanced invasion abilities, while overexpression reduced them (Figure 2F).

### 2.3 The Dysregulation of CD36 Impacted the Cellular Response to Oxidative Stress and Ferroptosis in TNBC Cells

**FIGURE 3:**
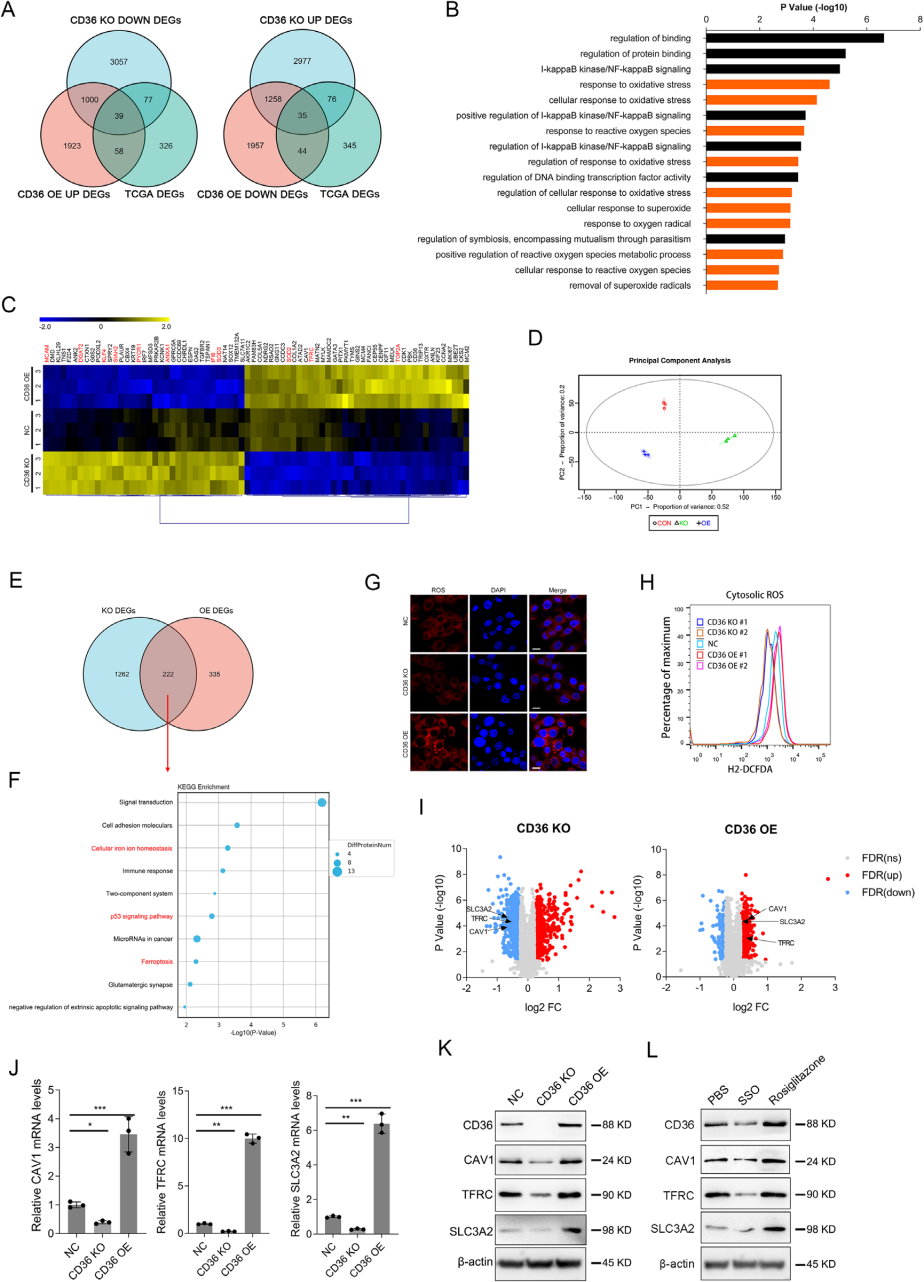
Multiomics analysis identifies CD36‐mediated oxidative stress and ferroptosis pathways. (A) Combined analysis of differentially expressed genes in CD36 alteration cells and the top 500 differentially expressed genes in TCGA‐BRCA. (B) GO enrichment analysis of proteomic results from CD36 alteration cells. (C) Heatmap analysis of common genes between differentially expressed genes in CD36 alteration cells and the top 500 differentially expressed genes in TCGA‐BRCA. (D) Grouping of samples for proteomics analysis. (E) Intersection analysis of significantly altered proteins in CD36 knockout and overexpressing cells. (F) KEGG enrichment analysis of commonly altered proteins after CD36 knockout and overexpression. (G) Immunofluorescence detection of ROS levels in CD36 alteration cells. (H) Flow cytometry analysis of ROS levels in CD36 alteration cells. (I) Volcano plot of significantly altered proteins in CD36 alteration cells. (J) Validation of mRNA levels of specific proteins CAV1, TFRC, SLC3A2 in MDA‐MB‐231 cells after CD36 alteration. (K) Western blot validation of specific proteins CAV1, TFRC, SLC3A2 in MDA‐MB‐231 cells after CD36 alteration. (L) Levels of specific proteins CAV1, TFRC, SLC3A2 in MDA‐MB‐231 cells treated with CD36 agonist rosiglitazone and CD36 inhibitor SSO.

To explore the impact of CD36 expression alterations on gene expression in TNBC cells, we performed RNA sequencing and proteomic analysis on TNBC cells with CD36 alteration. The transcriptomic analysis identified 2242 differentially expressed genes (DEGs) following CD36 alteration (Figure S2B). To understand the biological significance of these DEGs, the Gene Ontology (GO) clustering analysis was conducted. Our results revealed significant changes in pathways related to cell response to oxidative stress (Figure 3B), while Kyoto encyclopedia of genes and genomes (KEGG) pathway analysis demonstrated their concurrent enrichment in ferroptosis‐related pathways (Figure S2C). In addition, we intersected the DEGs identified following CD36 alterations with the top 500 most significant DEGs from the TCGA‐BRCA dataset. This intersection revealed 39 genes positively correlated with CD36 expression and 35 genes negatively correlated with CD36 expression (Figure 3A). Heatmap analysis of the identified 74 genes highlighted key genes, including MCAM, KLF4, SOD2, SOD3, and TOP2A, which are known to regulate intracellular ROS levels, suggesting that CD36 alterations may lead to disordered ROS levels in TNBC cells (Figure 3C). To further validate the impact of CD36 expression on ROS levels, we performed immunofluorescence and flow cytometry analyses. The results demonstrated that ROS levels decreased following CD36 knockout, whereas CD36 overexpression led to an increase in ROS levels (Figure 3G,H).

Proteomic analysis was conducted to gain a deeper understanding of the protein networks and functions in TNBC cells following CD36 expression changes. The results revealed significant differences in protein expression upon CD36 alteration (Figure 3D). GO enrichment analysis of the proteomic data indicated that CD36 expression significantly influenced processes of cell membrane structure and calcium and iron ion binding (Figure S2D). Through cross‐analysis of the significantly altered proteins identified following CD36 knockout and overexpression, we identified 222 proteins with significant expression changes (Figure 3E). To identify the enriched pathways and functional categories associated with these proteins, we performed KEGG enrichment analysis, which revealed that CD36 expression changes impact key pathways related to ferroptosis (Figure 3F). By integrating transcriptomic and proteomic data, we identified key proteins related to ferroptosis, including solute carrier family 3 member 2 (SLC3A2), transferrin receptor protein (TFRC), and CAV1, whose expression levels were influenced by changes in CD36 expression (Figure 3F,I). To further validate the relationship between CD36 expression and ferroptosis‐related proteins and genes, qPCR and western blotting analyses were performed. Our results demonstrated that CD36 overexpression led to a significant increase in the mRNA levels of CAV1, TFRC, and SLC3A2, while CD36 knockout resulted in a decrease in these mRNA levels. Additionally, CD36 overexpression or treatment with the CD36 agonist rosiglitazone (ROSI) significantly increased the protein levels of CAV1, TFRC, and SLC3A2. Conversely, CD36 knockout or treatment with the CD36 inhibitor sulfo‐N‐succinimidyl oleate (SSO) led to significant decrease in these protein levels (Figure 3J–L). These findings suggested that ferroptosis levels are positively correlated with CD36 expression in TNBC cells.

### 2.4 CD36 Increased Lipid Peroxidation Level

**FIGURE 4:**
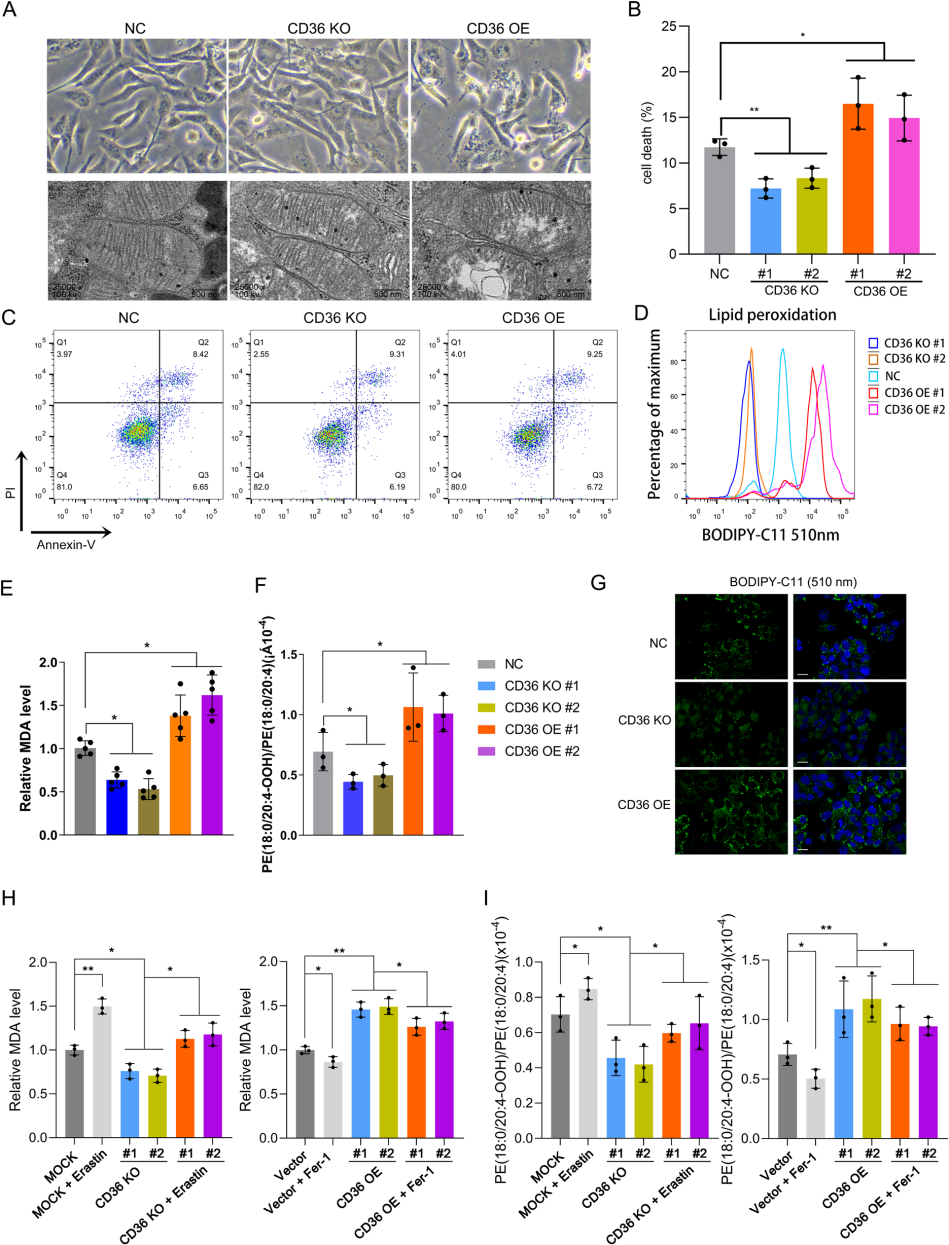
CD36 promotes ferroptosis by enhancing lipid peroxidation. (A) Morphological observation of mitochondrial damage in cells after CD36 alteration. (B) PI staining to detect the proportion of cell death in MDA‐MB‐231 cells after CD36 alteration. (C) Flow cytometry analysis of apoptosis levels in MDA‐MB‐231 cells with altered CD36 expression. (D) Flow cytometry analysis using BODIPY‐C11 (510 nm) to detect lipid peroxidation levels in cells. (E and F) Detection of MDA levels and 4‐OOH levels in MDA‐MB‐231 cells after CD36 alteration. (G) Confocal microscopy to observe fluorescence intensity after BODIPY‐C11 (510 nm) staining in cells. (H and I) Detection of MDA levels and 4‐OOH levels in MDA‐MB‐231 cells treated with ferroptosis agonists after CD36 knockout and ferroptosis inhibitors after CD36 overexpression. Data were analyzed using Student's *t*‐test and one‐way ANOVA (*p < 0.05, **p < 0.01, ***p < 0.001, ****p < 0.0001).

To further elucidate the relationship between CD36 levels and ferroptosis in TNBC cells, we utilized electron microscopy to observe cellular structure changes underlying CD36 alteration. The results revealed that CD36 overexpression increased mitochondrial damage (Figure 4A). Analysis using propidium iodide staining demonstrated that cell death levels decreased with CD36 knockout and increased with CD36 overexpression (Figure 4B). In contrast, flow cytometry analysis of apoptosis levels in cells with altered CD36 expression showed no significant difference in apoptosis levels (Figure 4C). This suggests that the cell death associated with changes in CD36 expression may be a nonapoptotic form of cell death.

Given that lipid peroxidation is a hallmark of ferroptosis, we assessed lipid peroxidation levels by measuring the levels of malondialdehyde (MDA), 4‐hydroxynonenal (4‐OOH), and BODIPY‐C11 (510 nm), which are key markers of lipid peroxidation. Flow cytometry and confocal microscopy were used to detect and visualize BODIPY‐C11 (510 nm) levels, revealing that lipid peroxidation levels increased with CD36 overexpression and decreased with CD36 knockout (Figure 4D,G). Additionally, MDA and 4‐OOH levels further confirming that these markers increased with CD36 overexpression and decreased with CD36 knockout (Figure 4E,F). These findings indicated that CD36 expression significantly influences lipid peroxidation levels, thereby affecting the ferroptosis process in TNBC cells.

To further validate CD36's role in regulating ferroptosis, we treated CD36 knockout MDA‐MB‐231 cells with the ferroptosis activator erastin and CD36‐overexpressing cells with a ferroptosis inhibitor. The results showed that MDA and 4‐OOH levels increased in CD36 knockout cells following erastin treatment and decreased in CD36‐overexpressing cells treated with the ferroptosis inhibitor (Figure 4H,I). These findings demonstrated that CD36 might induce lipid peroxidation and thereby upregulate ferroptosis levels in TNBC cells.

### 2.5 CD36 Positively Regulated CAV1 Expression

**FIGURE 5:**
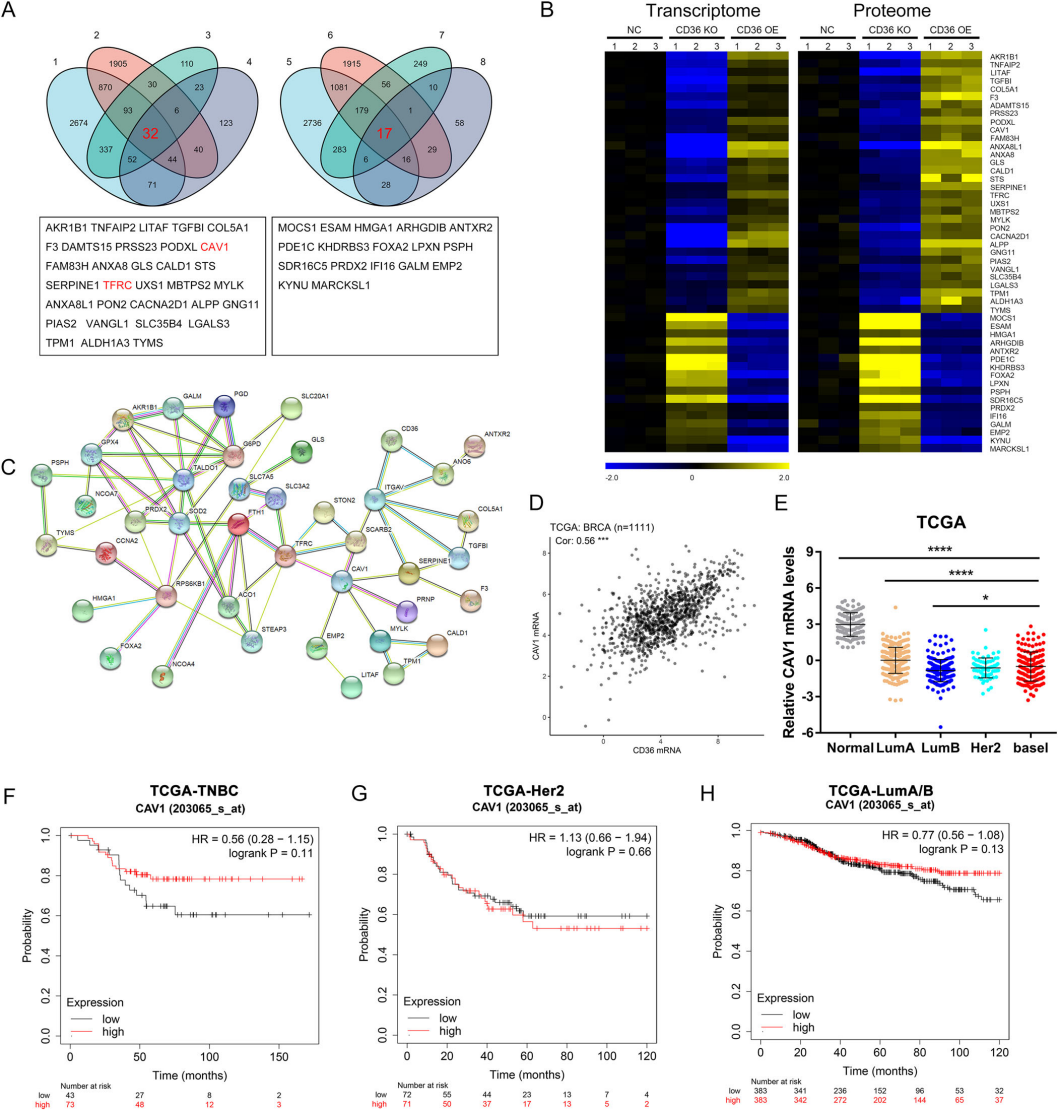
Integrated transcriptomic and proteomic analysis reveals CD36–CAV1 as a central axis in TNBC progression. (A) Venn diagram showing the combined analysis of transcriptome and proteome after altering CD36 expression. (1. Downregulated genes in the CD36 knockout group in the transcriptome results; 2. Upregulated genes in the CD36 overexpression group in the transcriptome results; 3. Downregulated genes in the CD36 knockout group in the proteome results; 4. Upregulated genes in the CD36 overexpression group in the proteome results; 5. Upregulated genes in the CD36 knockout group in the transcriptome results; 6. Downregulated genes in the CD36 overexpression group in the transcriptome results; 7. Upregulated genes in the CD36 knockout group in the proteome results; 8. Downregulated genes in the CD36 overexpression group in the proteome results). (B) Heatmap showing the common genes altered in the transcriptome and proteome of MDA‐MB‐231 cells after CD36 alteration. (C) Interaction network analysis of the common altered genes. (D) Correlation analysis of CAV1 and CD36 in TCGA‐BRCA cohorts. (E) CD36 mRNA levels of different subtypes in breast cancer, data were obtained from TCGA‐BRCA cohorts. (F) Kaplan–Meier survival curves showing the differences in CAV1 expression among Her2+ subtypes in the TCGA‐BRCA cohorts. (G) Kaplan–Meier survival curves showing the differences in CAV1 expression among different breast cancer subtypes in the TCGA‐BRCA cohorts. (H) Kaplan–Meier survival curves showing the differences in CAV1 expression among luminal A/B subtypes in the TCGA‐BRCA cohorts. Survival curves figures created with kmplot.com.

To further elucidate the mechanism by which CD36 regulates ferroptosis, a comprehensive analysis of transcriptomic and proteomic data was conducted on TNBC cells with altered CD36 expression. A total of 49 molecules were identified, with 32 showing a positive correlation with CD36 expression and 17 exhibiting a negative correlation (Figure 5A,B). An interaction network analysis of the coregulated proteins highlighted CAV1 as a key intermediary molecule connecting CD36 with ferroptosis‐related proteins (Figure 5C). To further validate the correlation between CD36 and CAV1, we analyzed data from TCGA‐BRCA database, which revealed a significant positive correlation between the expression levels of CAV1 and CD36 (Figure 5D). Moreover, statistical analysis of gene data from the TCGA database confirmed that the expression trend of CAV1 in breast cancer tissues was consistent with that of CD36, with CAV1 expression significantly lower in cancer tissues compared with normal tissues (Figure 5E). These findings confirmed with previous validation of CAV1 mRNA and protein levels that changed followed by CD36 alteration (Figure 3J,K). Interestingly, similar to CD36, CAV1 expression levels were positively correlated with patient prognosis in TNBC and luminal subtypes of breast cancer (Figure 5F,H), whereas no significant correlation was observed in HER2‐positive breast cancer subtype (Figure 5G). These results suggested that CAV1 might be a downstream molecule positively regulated by CD36 in TNBC cells.

### 2.6 CD36 Regulates Ferroptosis Through Modulation of CAV1 Expression

**FIGURE 6:**
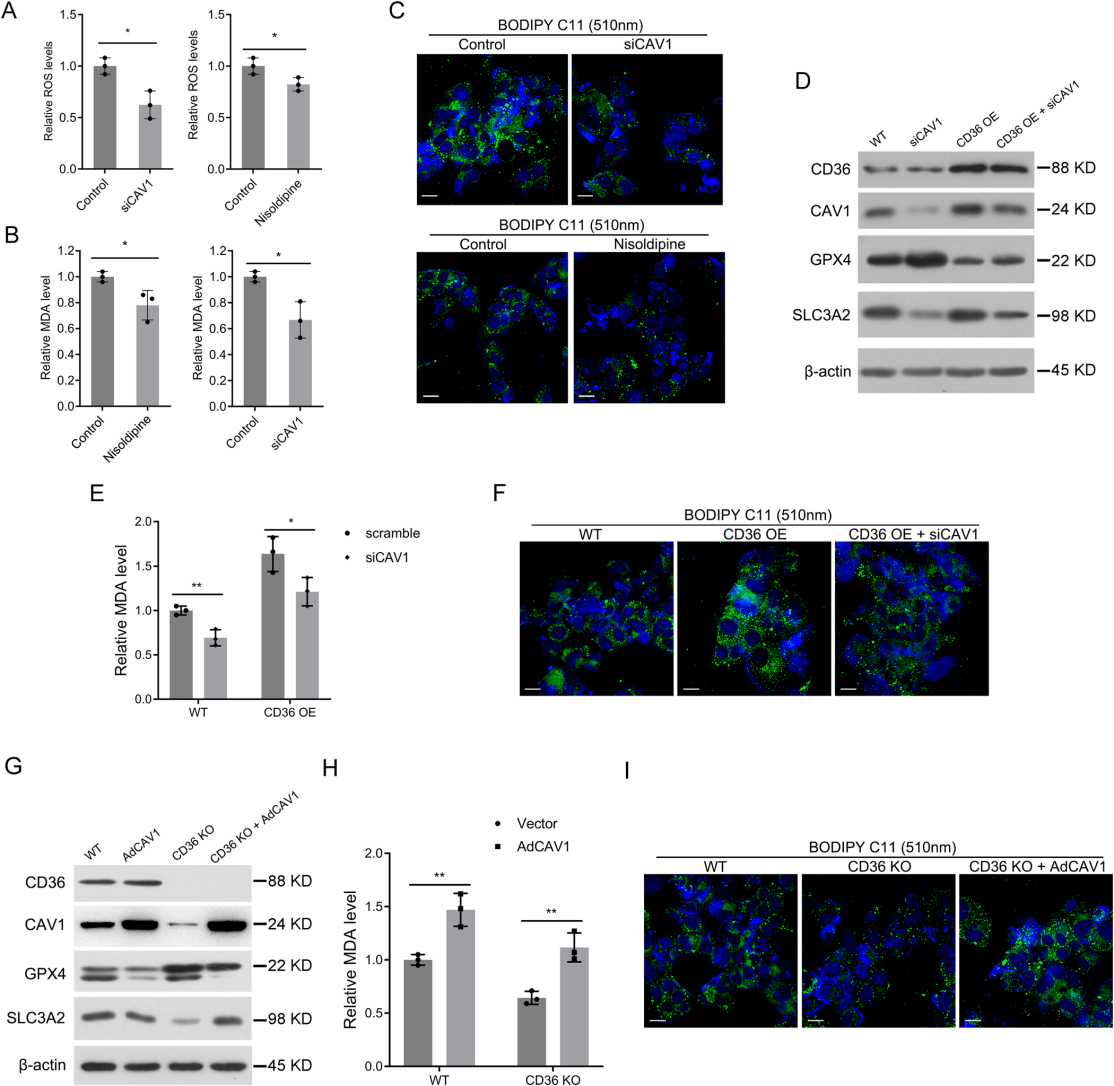
CD36 regulates ferroptosis via CAV1‐dependent lipid peroxidation. (A) MDA‐MB‐231 cells treated with siCAV1 or CAV1 inhibitor (nisoldipine) to detect intracellular ROS levels. (B) MDA‐MB‐231 cells treated with siCAV1 or CAV1 inhibitor (nisoldipine) to detect intracellular MDA levels. (C) Confocal microscopy observation of fluorescence intensity after BODIPY‐C11 (510 nm) staining. (D) Western blots of ferroptosis‐related proteins in MDA‐MB‐231 cells with CD36 overexpression and/or CAV1 silence, GAPDH was loaded as a control. (E) Detection of intracellular MDA levels in MDA‐MB‐231 cells with CD36 overexpression and/or CAV1 silence. (F) Observation of BODIPY‐C11 levels in MDA‐MB‐231 cells with CD36 overexpression and/or CAV1 silence. (G) Western blots of ferroptosis‐related proteins in MDA‐MB‐231 cells with CD36 knockout and/or CAV1 overexpression, GAPDH was loaded as a control. (H) Detection of intracellular MDA levels in MDA‐MB‐231 cells with CD36 knockout and/or CAV1 overexpression. (I) Observation of BODIPY‐C11 levels in MDA‐MB‐231 cells with CD36 knockout and/or CAV1 overexpression. Data were analyzed using Student's *t*‐test and one‐way ANOVA (*p < 0.05, **p < 0.01, ***p < 0.001, ****p < 0.0001).

CAV1 functions as an oxidative stress sensor, regulating the cellular response to oxidative stress and playing a role in ferroptosis regulation through FA metabolism. To confirm the relationship between CD36‐induced changes in cell ferroptosis levels and CAV1, experiments were conducted using siRNA to silence CAV1 expression and treating MDA‐MB‐231 cells with the CAV1‐specific inhibitor nisoldipine. Both CAV1 silencing and nisoldipine treatment led to reductions in intracellular ROS, MDA, and BODIPY‐C11 (510 nm) levels, as determined by ROS and MDA detection kits and immunofluorescence analysis (Figure 6A–C), indicated that CAV1 inhibition can reduce lipid peroxidation levels in TNBC cells. Next, CAV1 was knocked down in CD36‐overexpressing MDA‐MB‐231 cells, and ferroptosis‐related markers were detected using western blotting. The results demonstrated that the decrease of ferroptosis inhibitor GPX4 induced by CD36 overexpression was reversed upon CAV1 knockdown, while the increased level of the ferroptosis inhibitor SLC3A2 decreased followed by CAV1 knockdown (Figure 6D). Additionally, after silencing CAV1 in CD36‐overexpressing cells, measurements of MDA and BODIPY‐C11 (510 nm) levels indicated that CAV1 silencing effectively reversed the increase in MDA and lipid peroxidation levels caused by CD36 overexpression (Figure 6E,F).

Conversely, in CD36‐knockout MDA‐MB‐231 cells, CAV1 overexpression led to measurements of ferroptosis‐related factors, including SLC3A2 and GPX4, as well as MDA and lipid peroxidation levels, demonstrating that CAV1 overexpression could reverse the decrease in ferroptosis levels, MDA levels, and lipid peroxidation levels observed in these cells (Figure 6G–I). Thus, in MDA‐MB‐231 cells, CD36 regulated CAV1 expression and influenced ferroptosis through the CAV1 pathway.

### 2.7 CD36 Regulated CAV1 Expression by Modulating PPARγ Transcriptional Activity

**FIGURE 7:**
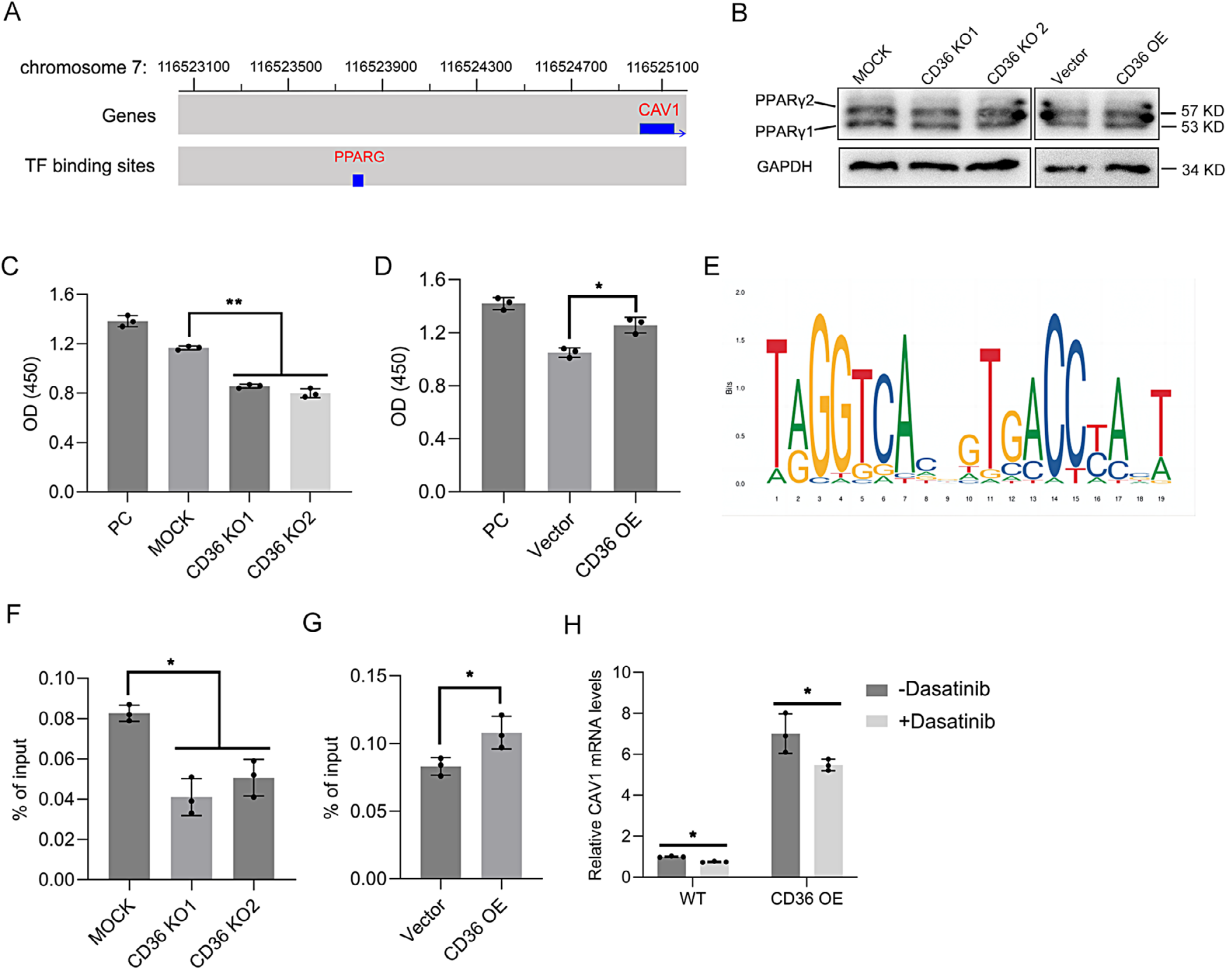
CD36 activates PPARγ to transcriptionally upregulate CAV1. (A) Predictive analysis of transcription binding sites of PPARγ on the promoter region of CAV1. (B) Western blots of PPARγ in MDA‐MB‐231 cells with CD36 alteration, GAPDH was loaded as a control. (C) Protein activity analysis of PPARγ with CD36 knockout. (D) Protein activity of PPARγ with CD36 overexpression. (E) Display of PPARγ binding sequences in the CAV1 promoter region obtained by JASPAR website. (F) ChIP‐qPCR assay to detect the changes in the binding level of PPAR in the CAV1 promoter region following CD36 knockout. (G) ChIP‐qPCR assay to detect the changes in the binding level of PPAR in the CAV1 promoter region following CD36 overexpression. (H) CAV1 mRNA levels in CD36 overexpression cells treated with the PPARγ inhibitor Dasatinib. Data were analyzed using Student's *t*‐test and one‐way ANOVA (*p < 0.05, **p < 0.01, ***p < 0.001, ****p < 0.0001).

Given previous results indicating that CD36 alters CAV1 mRNA levels, we hypothesized that CD36 might control CAV1 transcription and conducted a transcription factor prediction analysis on the CAV1 promoter region. Interestingly, PPARγ, a transcription factor whose activity is reportedly regulated by CD36, was predicted to bind with the CAV1 promoter region (Figure 7A). Although western blotting revealed no change in PPARγ protein levels in cells with altered CD36 expression (Figure 7B), further analysis demonstrated that CD36 alteration significantly affected PPARγ protein activity, with PPARγ activity decreasing after CD36 knockout and increasing following CD36 overexpression (Figure 7C,D). Using JASPAR, one PPARγ binding motif in the CAV1 promoter region was obtained (Figure 7E). To demonstrate CD36 alteration whether impact PPARγ’s transcriptional activity in regulation of CAV1, a chromatin immunoprecipitation (ChIP) assay using a PPARγ antibody was conducted. The results demonstrated a significant decrease in PPARγ binding to the CAV1 promoter region in CD36 knockout cells, whereas an increase in PPARγ binding levels in the CAV1 promoter was observed in CD36‐overexpressing cells (Figure 7F,G). To further verify this regulatory pathway, we treated CD36‐overexpressing cells with the PPARγ inhibitor dasatinib and subsequently observed the resulting CAV1 mRNA levels. Indeed, the increase in CAV1 mRNA levels induced by CD36 overexpression was reversed upon treatment with PPARγ inhibition (Figure 7H). These findings suggest that CD36 enhances CAV1 transcription by upregulating the transcriptional activity of PPARγ.

### 2.8 Pharmacological Treatment with CD36 Agonist Inhibited the Metastasis of TNBC Cells in Mice in Vivo

**FIGURE 8:**
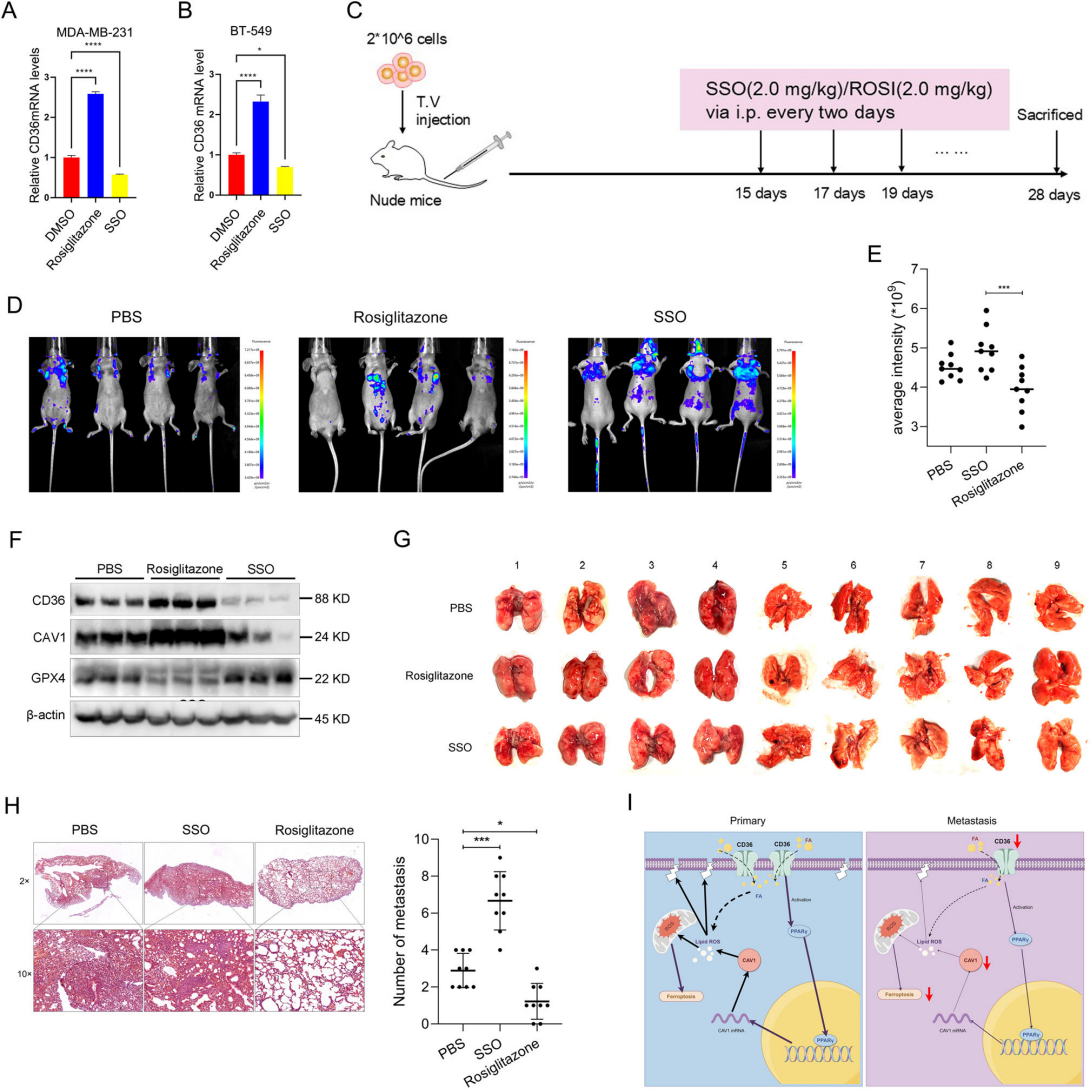
CD36 activation suppresses TNBC metastasis in vivo. (A) CD36 mRNA levels in MDA‐MB‐231 cells treated with the CD36 agonist rosiglitazone or CD36 inhibitor SSO. (B) CAV1 mRNA levels in MDA‐MB‐231 cells treated with the rosiglitazone or SSO. (C) Experimental procedure. (D and E) Fluorescence in vivo imaging to observe the overall metastasis of xenograft tumors in nude mice upon rosiglitazone or SSO treatment. (F) Western blots of CD36, CAV1, and GPX4 in lung metastatic tumors in nude mice. (G) Macroscopic observation of lung metastases in nude mice. (H) H&E staining of lung tissue sections in nude mice and statistics on the number of lung metastases in nude mice. (I) Schematic diagram summarizing the working model, figure created with BioRender.com. Data were analyzed using Student's *t*‐test and one‐way ANOVA (*p < 0.05, **p < 0.01, ***p < 0.001, ****p < 0.0001).

Building on our in vitro findings, we investigated the function of CD36 and its modulation by the agonist ROSI and inhibitor SSO in vivo using a nude mouse CDX model. Initially, in vitro experiments demonstrated that treatment with ROSI and SSO significantly increased and decreased CD36 and CAV1 mRNA levels in MDA‐MB‐231 cells, respectively (Figure 8A,B). In the in vivo metastasis model, mice received intraperitoneal injections of the CD36 agonist ROSI, the inhibitor SSO, or phosphate‐buffered saline (PBS) as a control, with treatments occurring every 2 days over a 2‐week period (Figure 8C). Fluorescence live imaging revealed that the ROSI intervention group exhibited significantly reduced metastasis compared with the control group, while the SSO intervention group showed a significant increase in metastasis (Figures 8D and S3A). Western blotting analysis of metastasis nodules from different pharmacological treatments revealed that the ROSI treatment resulted in increased levels of CD36 and CAV1, along with decreased levels of the ferroptosis inhibitor GPX4, whereas the SSO treatment exhibited decreased levels of CD36 and CAV1, accompanied by an increase in GPX4 (Figure 8F). Gross observation and hematoxylin and eosin (HE) staining of lung metastases in mice demonstrated that ROSI administration reduced the number of tumor nodules, while SSO administration increased the number of tumor nodules (Figure 8G,H), thereby confirming the in vivo antimetastatic effect of CD36 agonists on TNBC cells.

Given the role of CD36 in enhancing ferroptosis through the transcriptional upregulation of CAV1 in TNBC, we further investigated the potential of ferroptosis activators to effectively inhibit TNBC tumor metastasis in the absence of CD36. Stable CD36 knockdown MDA‐MB‐231 cells were injected into the tail vein of nude mice. After 2 weeks, the mice were randomly assigned to receive intraperitoneal injections of PBS or the ferroptosis activator erastin every 2 days for a total of five cycles. Nude mouse CDX model was set up using stable CD36 knockdown MDA‐MB‐231 cells, and mice received intraperitoneal injections of ferroptosis agonist erastin or PBS. The results demonstrated that the erastin treatment group exhibited significantly reduced liver and lung metastasis compared with the control group (Figure S3B–D). Western blotting analysis of metastasis nodules revealed an increase in CAV1 levels and a decrease in the levels of the ferroptosis inhibitors GPX4 and nuclear receptor coactivator 4 (NCOA4) after erastin treatment (Figure S3E). The findings suggested that enhancing CD36 levels and promoting ferroptosis could serve as a viable therapeutic strategy for treating TNBC. This approach underscores the sensitivity of TNBC cells to ferroptosis modulation, highlighting its potential as a targeted treatment avenue.

## 3 Discussion

The present study demonstrated that CD36 is generally downregulated in human TNBC, highlighting its potential role in the disease's progression. Our findings revealed a positive correlation between CD36 expression levels and the OS rate of TNBC patients. This suggests that CD36 plays a significant role in the prognosis of TNBC and may function as a tumor suppressor in this cancer subtype. These insights underscore the importance of CD36 in TNBC and its potential as a therapeutic target.

Recent evidence increasingly supports the notion that cancer is primarily a metabolic disease, characterized by disordered glucose, lipid, and amino acid metabolism within cancer cells. CD36, a FA transporter, plays a pivotal role in lipid metabolism and is implicated in various metabolic diseases [36]. Despite its well‐established function, the roles of CD36 in different tumors are contradictory, underscoring the complexity of its involvement in tumor biology. For instance, CD36 overexpression in oral cancer cells significantly stimulates lymph node metastasis and CD36+ cancer‐associated fibroblasts create an immunosuppressive microenvironment in hepatocellular carcinoma to promote tumor progression, suggesting oncogenic roles for CD36 [10, 37]. Conversely, CD36 acts as a tumor suppressor and inhibits aerobic glycolysis by promoting GPC4 ubiquitination and inhibiting β‐catenin/c‐myc signaling in colorectal cancer [16]. In our study, analysis of the TCGA database and CD36 protein staining on tissue microarrays revealed that CD36 is downregulated in TNBC, with its expression level significantly negatively correlated with the prognosis of this tumor type. These findings confirm the tumor suppressor role of CD36 in TNBC, and consistent with the prior study in colorectal cancer.

The paradoxical roles of CD36 in breast cancer—exhibiting both pro‐ and anticarcinogenic effects—warrant careful interpretation of its subtype‐specific functions [8, 9, 10, 11, 12, 13]. Our data demonstrate that CD36 is consistently downregulated across breast cancer subtypes, with the most pronounced suppression in TNBC, where reduced expression correlates with advanced disease stages and poorer prognosis. Functional studies in TNBC cells further revealed that CD36 inhibits proliferation, migration, and invasion, aligning with its proposed tumor‐suppressive role in this subtype. However, these findings appear to conflict with reports implicating CD36 in promoting metastasis in luminal or HER2‐positive breast cancers via FA uptake and metabolic reprogramming [38, 39]. This discrepancy suggests that CD36's role may be context dependent, influenced by subtype‐specific molecular landscapes. For instance, TNBC's unique metabolic demands, driven by glycolytic dominance rather than lipid dependency, might render CD36 loss advantageous for tumor progression, whereas lipid‐rich luminal/HER2+ microenvironments could exploit CD36‐mediated FA transport to fuel metastatic growth. Additionally, posttranslational modifications or interactions with subtype‐specific signaling pathways (e.g., EGFR in TNBC vs. HER2 in HER2+ tumors) may differentially regulate CD36's downstream effects. Importantly, our survival analysis highlights that CD36's prognostic relevance is restricted to TNBC, further supporting subtype‐specific functionality. Future studies should delineate how CD36 interfaces with subtype‐defining pathways and explore whether its tumor‐suppressive effects in TNBC involve mechanisms beyond lipid metabolism, such as immune modulation or anoikis resistance. These insights will clarify CD36's dual roles and inform its potential as a therapeutic target tailored to breast cancer subtypes.

Oxidative stress, a condition resulting from an imbalance between the production of ROS and their neutralization by antioxidants, plays a significant role in cellular physiology and pathology [40]. ROS serve dual functions within cells: they act as signaling molecules in normal physiological processes but can also cause cellular damage and death when present in high concentrations. In the context of diabetic nephropathy, CD36 has been implicated in mediating ROS production, promoting the activation of the NOD‐like receptor family pyrin domain containing 3 (NLRP3) inflammasome in renal tubular epithelial cells, inhibiting mitochondrial FA oxidation, and stimulating mitochondrial ROS production [41]. Additionally, studies have demonstrated that aldehyde dehydrogenase 2 can reduce the uptake of oxidized low‐density lipoprotein by macrophages by downregulating CD36 expression, which in turn inhibits NF‐κB activation and NLRP3 expression, leading to a decrease in inflammatory responses [42]. Our transcriptomic analysis identified numerous oxidative stress‐related genes whose expression is modulated by CD36. Furthermore, we directly observed alterations in ROS levels in cells with CD36 overexpression and knockout, thereby reinforcing the role of CD36 in the regulation of ROS.

ROS can induce lipid peroxidation, and lipid peroxides generated in free radical chain reactions can decompose into reactive products, such as MDA and 4‐hydroxy‐2‐nonenal, which cause cell membrane damage and can react with proteins and DNA, leading to cell dysfunction and genetic mutations. The iron‐dependent accumulation of lipid ROS is a hallmark of ferroptosis [43, 44]. As a key participant in FA metabolism, CD36 is closely linked to lipid peroxidation levels, a critical inducer of ferroptosis. Consequently, we hypothesized that CD36 might regulate ferroptosis in TNBC. Our findings support the hypothesis that CD36 significantly regulates ferroptosis, and we discovered that the expression of CAV1, a vital membrane protein that reported in regulation of ferroptosis, varies with CD36 levels. CAV1 acts as a sensor of oxidative stress, regulating the cellular response to oxidative stress, and has been implicated in the regulation of ferroptosis through FA metabolism [45, 46, 47]. By pharmacologically and genetically manipulating the expression of CD36 and CAV1, we observed corresponding changes in cellular ROS and lipid peroxidation levels, suggesting that CD36‐induced ferroptosis is mediated through CAV1 expression.

In our investigation into the regulatory mechanisms of CD36 on CAV1 expression, we identified a significant role for the transcription factor PPARγ. Previous studies have established that CD36 influences PPARγ activity, which in turn affects various physiological processes, including atherosclerosis, liver cholesterol biosynthesis, and adipose mitochondrial biogenesis [48]. Specifically, CD36, in conjunction with GHRP peptides, modulates downstream PPARγ pathways [49, 50, 51]. Building on this foundation, we discovered a PPARγ binding site within the CAV1 promoter region, offering a mechanistic explanation for the regulation of CAV1 by CD36 through PPARγ. This finding enhances our understanding of the intricate molecular interactions governing lipid metabolism and cellular responses in various pathological contexts.

Over the past decade, substantial evidence has emerged indicating that malignant tumor cells are particularly sensitive to ferroptosis, and that inducing this form of cell death can effectively inhibit tumor growth [34]. Despite significant research efforts, there remains a lack of highly effective targeted therapies for TNBC [52]. Inspired by the work of Ding et al. on inducing colorectal cancer cell death through AAV–CD36 knockdown, we explored the potential of targeting CD36 in TNBC therapy. Our study confirmed that manipulating CD36 expression can significantly alter the growth and metastasis of TNBC cells. This was achieved through various approaches, including AAV–CD36 overexpression and the use of CD36 agonists in combination with ferroptosis activators, both in cell culture and in nude mouse models. These findings provide robust theoretical support for targeting CD36 as a novel therapeutic strategy for TNBC.

## 4 Limitation

Although this study confirms the critical role of CD36 in ferroptosis induction and tumor suppression in TNBC, several important limitations warrant acknowledgment. First, the current lack of highly specific CD36‐targeted pharmacological agents (agonists and inhibitors) hinders precise modulation of CD36 activity in vivo. This paucity of key molecular tools restricts our ability to conduct a comprehensive evaluation of CD36‐targeted therapeutic strategies. Second, the molecular mechanisms underpinning the CD36–PPARγ–CAV1 regulatory axis require further elucidation through more systematic genetic and biochemical interrogation. Future studies employing rigorous epistasis analyses and pathway validation are necessary to fully delineate this signaling network. Third, the experimental design utilized a shared wild‐type control group for both the CD36‐knockout and CD36‐overexpression models. While the observed contrasting phenotypes robustly support a cell‐autonomous role for CD36 in the assessed phenotypes, we acknowledge the possibility that subtle differences arising from clonal variation or off‐target effects associated with the genetic manipulations could contribute to the results. Future investigations utilizing inducible expression systems or isogenic control lines specific to each genetic perturbation would strengthen causal attribution of the observed effects to CD36 modulation. Addressing these limitations will be crucial for enhancing the translational potential of CD36‐targeted therapies for TNBC.

## 5 Conclusions

In summary, our study revealed that CD36 expression is decreased in TNBC and that higher CD36 expression correlates with improved prognosis in TNBC patients, suggesting that CD36 may function as a tumor suppressor in this subtype of breast cancer. Mechanistically, CD36 exerts its tumor‐suppressive effect by regulating the expression and activity of CAV1 via PPARγ, which increases intracellular lipid peroxidation and promotes ferroptosis in TNBC cells (Figure 8I). Additionally, we validated the therapeutic potential of targeting the CD36–CAV1–ferroptosis pathway in TNBC using animal models. These findings strongly support the role of CD36 as a TNBC suppressor and suggest that its expression level may serve as an early biomarker for assessing the risk of malignant transformation and a potential target for TNBC intervention.

## 6 Materials and Methods

### 6.1 TCGA Data Acquisition and Processing

The mRNA expression and corresponding clinical information for breast cancer analyzed in this study were downloaded from TCGA database via the Genomic Data Commons Data Portal. The dataset was accessed and downloaded in 2019, which was prior to the implementation of the data access restrictions.

### 6.2 Clinical Samples

Breast cancer tissues and corresponding normal breast tissues (10 sets) were obtained from Southwest Hospital, Army Medical University. These samples were utilized for western blotting analysis. Additionally, tissue microarray samples for IHC were procured from Shanghai Outdo Biotech Co., Ltd.

### 6.3 Animal Models

Four‐week‐old NOD‐Scid mice were housed under specific pathogen‐free conditions with controlled temperature and humidity at the Animal Experiment Center of Army Medical University. The mice had free access to food and water. After a 1‐week acclimation period, TNBC models were established. All animal experiments adhered to the ethical guidelines of Army Medical University.

TNBC cell suspensions, collected during the logarithmic growth phase, were injected into the mice via the tail vein at a concentration of 1 × 10^7^ cells per mouse. The colonization of TNBC cells was monitored using an in vivo imaging system on days 15 and 30 postinjection. The mice were euthanized, and samples were collected 30 days after the injection.

### 6.4 Cell Culture

Breast cancer cell lines were obtained from the Experimental Center of Xiangya School of Medicine, Central South University. Cells were cultured in RPMI 1640 medium supplemented with 10% fetal bovine serum, 1% penicillin–streptomycin, and 1% glutamine at 37°C in a 5% CO_2_ atmosphere. Cells were passaged using 0.25% trypsin. Lentivirus vectors were purchased from HanBio Co., Ltd (located in Shanghai, China). The CD36 shRNA (5′‐GGACCATTGGTGATGAGAAGGCAAACATG‐3′) and CAV1 shRNA (5′‐GATTGATCTGGTCAACCGC‐3′) were used. CD36 knockout cell lines was constructed by HanBio Co., Ltd, using CRISPR–Cas9 technology.

### 6.5 HE Staining

Liver tissues were fixed in 10% neutral formalin for 48 h, followed by routine dehydration, paraffin embedding, and sectioning. Sections were deparaffinized with xylene, hydrated, stained with hematoxylin, differentiated with 0.5% hydrochloric acid alcohol, and counterstained with 0.5% eosin solution. After dehydration and clearing, sections were mounted with neutral balsam and observed under a light microscope for morphological changes.

### 6.6 Western Blotting Analysis

Tissue samples were lysed in buffer and homogenized. After centrifugation at 10700×g for 10 min at 4°C, the supernatant was collected, and protein concentration was determined using the bicinchoninic acid assay. Proteins were separated by 10% sodium dodecyl sulfate‐polyacrylamide gel electrophoresis and transferred onto polyvinylidene difluoride membranes. Membranes were blocked with 5% skim milk for 1 h, followed by overnight incubation with the primary antibody at 4°C. After washing with tris‐buffered saline with tween (TBST), membranes were incubated with an HRP‐labeled secondary antibody for 1 h and washed again with TBST. Protein bands were visualized using the electrochemiluminescence, with glyceraldehyde‐3‐phosphate dehydrogenase (GAPDH) serving as the loading control. Grayscale analysis was performed using Image J to quantify the relative expression levels of target proteins.

### 6.7 Flow Cytometry

For apoptosis analysis, cells were stained using the Annexin V‐FITC/DAPI apoptosis detection kit. For cell marker analysis, cells were resuspended in 1 mL of PBS and stained with fluorescent antibodies according to the manufacturer's instructions. Flow cytometry analysis was conducted immediately thereafter.

### 6.8 Reverse Transcription‐Quantitative Polymerase Chain Reaction

Total RNA was extracted using TRIzol reagent (Thermo Fisher Scientific, Waltham, MA, USA). Reverse transcription was performed using the Takara reverse transcription kit (Takara, Kusatsu, Shiga, Japan). RT‐qPCR was conducted on a real‐time PCR detection system using Bimake SYBR Green qPCR Master Mix (Houston, TX, USA). Ct values of target genes were normalized to ACTB, and gene expression was analyzed using the 2^−ΔΔCt^ method. Primer sequences for all genes are listed in Supplementary Table S1.

### 6.9 siRNA and Cell Transfection

MDA‐MB‐231 cells were transfected with siRNA targeting CAV1 or a nontargeting siRNA pool using Lipofectamine RNAiMAX (Life Technologies) as per the manufacturer's instructions. Cells were lysed in RIPA buffer 72 h following transfection.

### 6.10 Plasmid DNA Construction, Lentivirus Packaging, and Generation of Stable Cell Lines

The pReceiver‐Lv155 Puro plasmid was obtained from FunenGen.Lto (Guangzhou, China). For CD36 or CAV1 overexpression, the coding sequences were inserted into the MCS domain of the expression plasmid. LentiCRISPR‐v2, pMD2.G, and psPAX2 plasmids were obtained from Hanbio Ltd. (Shanghai, China). The sgRNA sequences, targeting CD36 for CRISPR/Cas9‐mediated gene editing, were obtained from the E‐CRISP website (http://www.e‐crisp.org/ECRISP/designcrispr.html, accessed on August 12, 2021). Lentiviral vectors were cotransfected with 2 µg of psPAX2 and 1 µg of pMD2.G into HEK293T cells using lipo8000 reagent (Beyotime, Shanghai, China). MDA‐MB‐231 cells were infected with CD36 sgRNA virus, isolated into single clones in a 96‐well plate, and expanded. CD36 knockout was confirmed through western blotting analysis and DNA sequencing.

### 6.11 RNA Sequencing

Cells were collected for RNA sequencing analysis using Trizol reagent (Invitrogen, Carlsbad, CA, USA). cDNA libraries were prepared and sequenced using the Illumina Novaseq 6000 platform at Beijing Qingke Biotechnology Co., Ltd. (Beijing, China).

### 6.12 Lipid ROS Detection

Lipid ROS levels were assessed using the BODIPY C11 kit (Thermo Fisher Scientific). Cells were seeded in six‐well plates at 5 × 10^4^ cells per well and cultured for 24 h. Following staining with the C11‐BODIPY probe, the cells were observed using a laser scanning confocal microscope (Olympus), with oxidized BODIPY (O‐BODIPY) visualized using excitation/emission wavelengths of 488/510 nm.

### 6.13 Detection of Hydroperoxy‐PE Species

Lipid and oxidized metabolite analysis was conducted using the Thermo Scientific Orbitrap Fusion Lumos Tribrid mass spectrometer. LC/MS data were evaluated using the Compound Discoverer software package (Thermo Fisher Scientific) following the manufacturer's instructions.

### 6.14 The Electrophoretic Mobility Shift Assay

The light shift chemiluminescent electrophoretic mobility shift assay kit (Thermo Fisher Scientific, 20148) was used to detect activity of PPARγ interaction with PPAR response element (PPRE). The sequences of PPRE consensus oligonucleotides were as follows: forward, 5′‐CAAAACTAGGTCAAAGGTCA‐3′; reverse, 5′‐GTTTTGATCCAGTTTCCAGT‐3′.

### 6.15 Transmission Electron Microscopy Detection of Mitochondrial Morphological Changes in TNBC Cells

Following treatment, the cells were fixed with 2.5% glutaraldehyde at room temperature for 2 h, followed by 1% osmium tetroxide for 1 h. Samples were then dehydrated, infiltrated with propylene oxide, and embedded in resin. After sectioning, double staining with uranyl acetate and lead citrate was performed. Mitochondrial morphology was observed and photographed using transmission electron microscopy.

### 6.16 Immunohistochemistry

Paraffin‐embedded tissues were sectioned at 4 µm thickness, deparaffinized, and subjected to antigen retrieval using a pressure cooker. Sections were blocked with goat serum, and endogenous peroxidase activity was neutralized with 3% hydrogen peroxide. Primary antibodies were applied overnight at 4°C. Protein visualization was achieved using the ABC peroxidase staining kit (Thermo Fisher Scientific) and DAB detection kit (ZSGB‐BIO, Beijing, China), followed by secondary antibody incubation.

### 6.17 Chromatin Immunoprecipitation

ChIP was performed using a ChIP detection kit (Wanleibio, Liaoning, China). Cells were crosslinked with 1% formaldehyde, and genomic DNA was fragmented by sonication. Immunoprecipitation was performed using PPARγ ChIP‐grade or normal rabbit IgG antibodies, followed by incubation with protein A + G beads. DNA–protein complexes were decrosslinked, and DNA was extracted and analyzed through qRT‐PCR.

### 6.18 Statistical Analysis

Data analysis was performed using GraphPad Prism 8.3.0. Results were expressed as means ± standard deviation. Differences between groups were analyzed using unpaired or paired Student's *t*‐tests. The Pearson correlation coefficient was used to assess relationships among variables.

## Author Contributions

Y. Z. and J. X. designed research and supervised the experimental work; X. J. W., Z. H. P., Y. W. and T. T. Z. performed all experiments; X. N. T., W. T. Y., and Y. Q. Z. contributed clinical samples; X. J. W., Z. H. P., X. W. Q., J. X., and Y. Z. analyzed the data and interpreted the results; X. J. W., Z. H. P., and Y. W. wrote the manuscript; X. W. Q. and Y. Z. revised the manuscript. All authors reviewed and approved the manuscript before submission.

## Funding

The present study was funded by the National Science Foundation for Young Scientists of China (Grant no. 81802665), Outstanding Young Talent Pool of Army Medical University (XZ‐2019‐505‐011), Chongqing Graduate Research and Innovation Project (CYB23293), Chongqing Major Medical Research Program (Joint Program of Chongqing Municipal Health Commission and Science and Technology Bureau) (Grant no. 2024DBXM001), Chongqing Clinical Diagnosis and Treatment Center of Breast Cancer (Grant no. 425Z2a1), Military Key Clinical Specialty (Grant no. 51561Z23612), and National Science Foundation for Young Scientists of China (Grant no. 82203057).

## Ethics Statement

All animal experiments and clinical trials involved were conducted in accordance with the ethical policies and procedures approved by the Ethics Committee of the First Affiliated Hospital of Army Medical University (Approval No. (A) KY2023068).

## Conflicts of Interest

The authors declare no conflicts of interest.

## Supporting information




**Table 1**. list of qPCR primers.
**Figure S1**: Expression levels of CD36 in tumors and its correlation with the prognosis of different types of breast cancer. (A) Expression of CD36 in different tumors from the TCGA database. (B) Kaplan–Meier survival curves in overall breast cancer, data sourced from KM plotter. (C) Kaplan–Meier survival curves in TNBC, data sourced from KM plotter. (D) Kaplan–Meier survival curves in HER2 positive breast cancer, data sourced from KM plotter. (E and F) Kaplan–Meier survival curves in LumA/B breast cancer, data sourced from KM plotter.
**Figure S2**: Transcriptomic and proteomics analysis of MDA‐MB‐231 cells after CD36 expression alteration. (A) Analysis of CD36 protein levels in different breast cancer cells. (B) Heatmap analysis of differentially expressed genes from transcriptome sequencing of CD36 knockout/overexpression cells. (C) KEGG enrichment analysis of coaltered genes following CD36 knockout and overexpression. (D) GO enrichment analysis of proteomics results in CD36 knockout and overexpressing cells.
**Figure S3**: In vivo animal experiments. (A) Fluorescence in vivo imaging to observe the overall metastasis of xenograft tumors in nude mice upon rosiglitazone or SSO treatment. (B) Macroscopic observation of lung tissue sections in nude mice. (C) Using fluorescence in vivo imaging to observe the effect of the ferroptosis activator (erastin) on the overall metastasis of xenograft tumors in nude mice. (D) H&E staining of lung tissue sections in nude mice. (E) Using Western blot to detect the effect of ferroptosis activator (erastin) on the protein levels of CD36, CAV1, GPX4, and NCOA4 in nude mouse metastases.

## Data Availability

All data generated or analyzed during this study are included in this manuscript (and its supplementary information files).
