# Supplementary material for: CD36 Inhibits Triple‐Negative Breast Cancer Progression by Transcriptionally Upregulating Caveolin‐1 and Promoting Lipid‐Reactive Oxygen Species‐Related Ferroptosis
Source: MedComm (2020). 2025 Nov 18;6(12):e70493. doi: 10.1002/mco2.70493 (PMC12627234; doi:10.1002/mco2.70493)
Supplement: Supplementary file 1 — Table 1. list of qPCR primers. Figure S1: Expression levels of CD36 in tumors and its correlation with the prognosis of different types of breast cancer. (A) Expression of CD36 in different tumors from the TCGA database. (B) Kaplan–Meier survival curves in overall breast cancer, data sourced from KM plotter. (C) Kaplan–Meier survival curves in TNBC, data sourced from KM plotter. (D) Kaplan–Meier survival curves in HER2 positive breast cancer, data sourced from KM plotter. (E and F) Kaplan–Meier survival curves in LumA/B breast cancer, data sourced from KM plotter. Figure S2: Transcriptomic and proteomics analysis of MDA‐MB‐231 cells after CD36 expression alteration. (A) Analysis of CD36 protein levels in different breast cancer cells. (B) Heatmap analysis of differentially expressed genes from transcriptome sequencing of CD36 knockout/overexpression cells. (C) KEGG enrichment analysis of coaltered genes following CD36 knockout and overexpression. (D) GO enrichment analysis of proteomics results in CD36 knockout and overexpressing cells. Figure S3: In vivo animal experiments. (A) Fluorescence in vivo imaging to observe the overall metastasis of xenograft tumors in nude mice upon rosiglitazone or SSO treatment. (B) Macroscopic observation of lung tissue sections in nude mice. (C) Using fluorescence in vivo imaging to observe the effect of the ferroptosis activator (erastin) on the overall metastasis of xenograft tumors in nude mice. (D) H&E staining of lung tissue sections in nude mice. (E) Using Western blot to detect the effect of ferroptosis activator (erastin) on the protein levels of CD36, CAV1, GPX4, and NCOA4 in nude mouse metastases. [file MCO2-6-e70493-s001.pdf]

CD36 inhibits triple-negative breast cancer progression by transcriptionally upregulating Caveolin-1 and promoting lipid-reactive oxygen species-related ferroptosis

Xiujuan Wu[1#], Yan Wang[1#], Zaihui Peng[1#], Tingting Zhao[1], Xuanni Tan[1], Wenting Yan[1], Yuqin Zhou[1], Jie Xia[2,3*], Xiaowei Qi[1*], Yi Zhang[1*]

1 Department of Breast and Thyroid Surgery/Key Laboratory of Chongqing Health Commission for Minimally Invasive and Precise Diagnosis and Treatment of Breast Cancer, Southwest Hospital, Army Medical University, Chongqing 400038, China.

2 Key Laboratory of Molecular Biology for Diseases (Ministry of Education), Institute for Viral Hepatitis, Department of Infectious Diseases, The Second Affiliated Hospital, Chongqing Medical University, Chongqing 400016, China.

3 Western(Chongqing) Collaborative Innovation Center for Intelligent Diagnostics and Digital Medicine, Chongqing National Biomedicine Industry Park, No. 28 Gaoxin Avenue, High-tech Zone, Chongqing 401329, China

*Corresponding Author: Yi Zhang, yzhang@tmmu.edu.cn; Xiaowei Qi, qxw9908@tmmu.edu.cn; Jie Xia, xiajie@cqmu.edu.cn

# These authors contributed equally to this work and shared first authorship.

The authors declare no potential conflicts of interest

Table 1. list of qPCR primers

Genes	primers	
	Forward	Reverse
CD36	5´-TGATGAACAGCAGCAACA-3´	5´-CACAGCCAGATTGAGAACT-3´
CAV1	5´-CTTCACCACCTTCACTGT-3´	5´-GGAATAGACACGGCTGAT-3´
SLC3A2	5´-TCACAAGAACCAGAAGGATG-3´	5´-AGTAGAACCAGAATCAGACAG-3´
TFRC	5´-GGACAGCACAGACTTCAC-3´	5´-GCCTTACTATACGCCACATA-3´

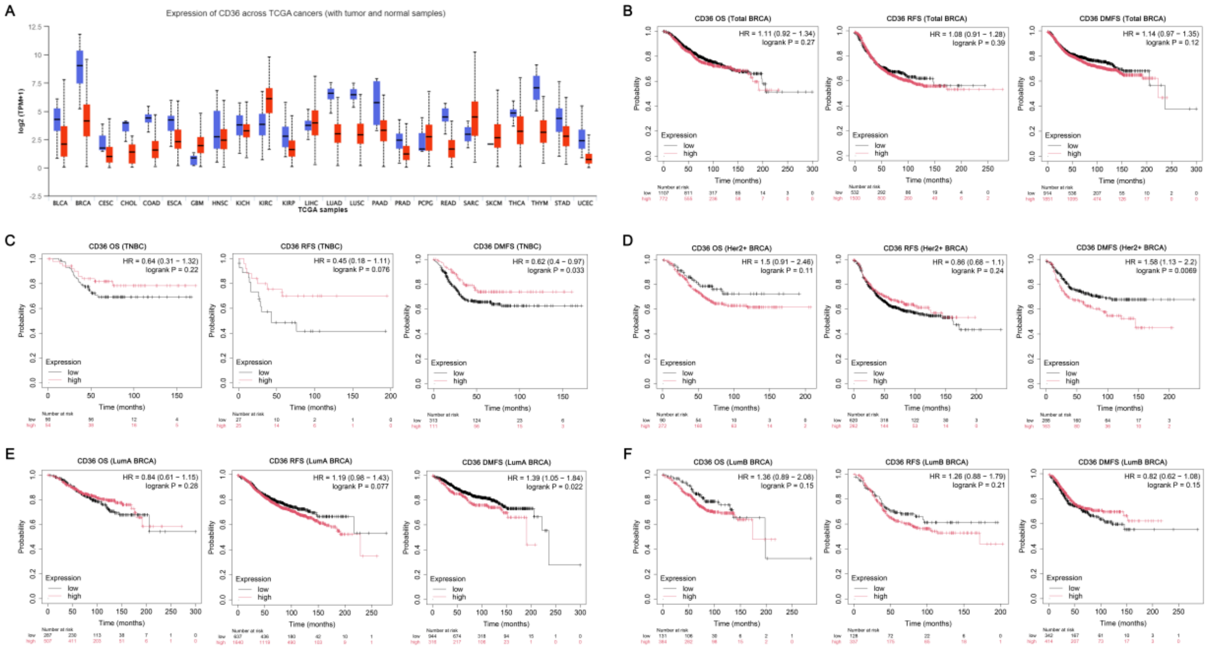

Figure S1: Expression levels of CD36 in tumors and its correlation with the prognosis of different types of breast cancer. (A) Expression of CD36 in different tumors from the TCGA database. (B) Kaplan-Meier survival curves in overall breast cancer, data sourced from KM plotter. (C) Kaplan-Meier survival curves in TNBC, data sourced from KM plotter. (D) Kaplan-Meier survival curves in HER2 positive breast cancer, data sourced from KM plotter. (E and F) Kaplan-Meier survival curves in LumA/B breast cancer, data sourced from KM plotter.

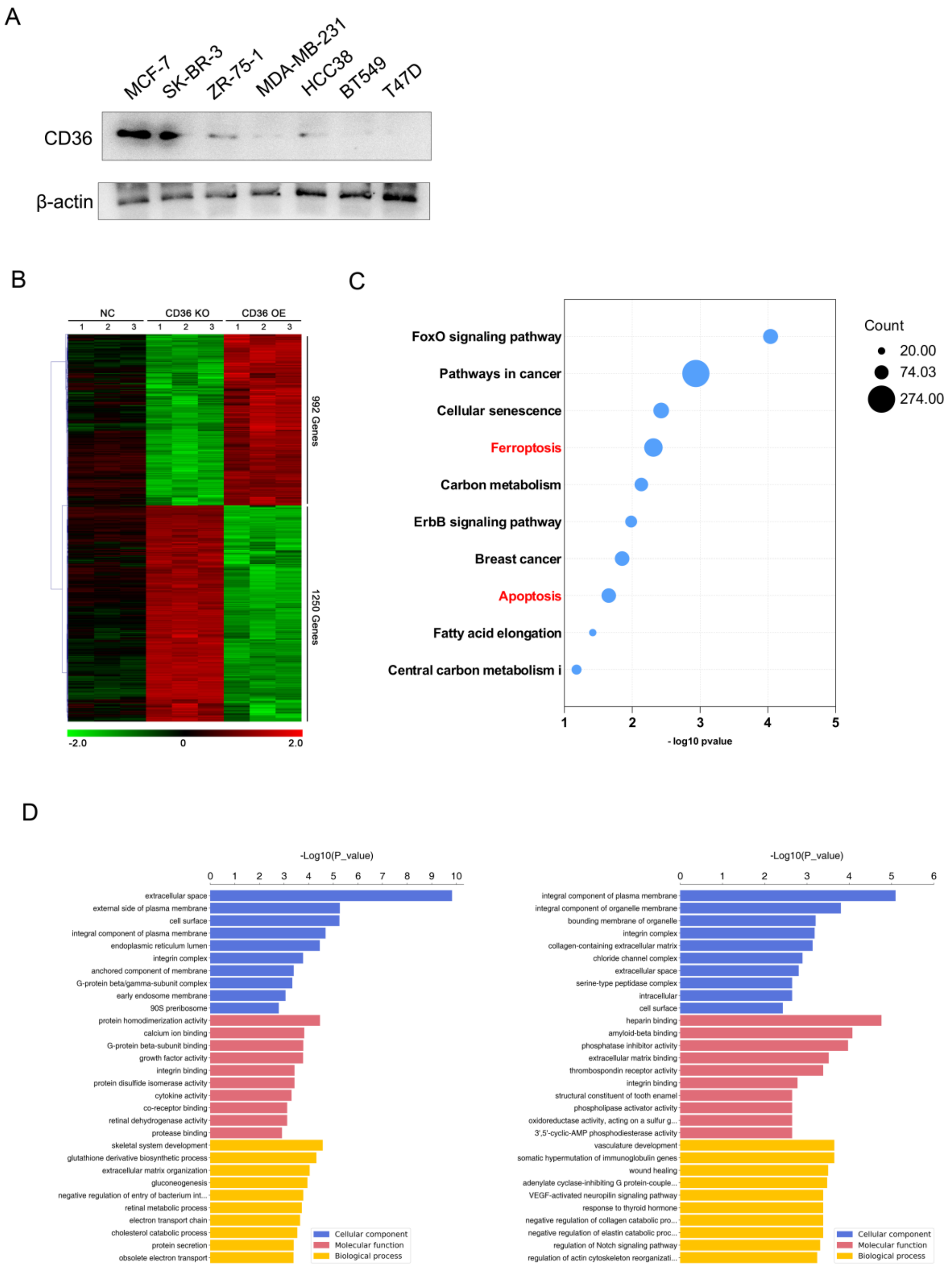

Figure S2: Transcriptomic and proteomics analysis of MDA-MB-231 cells after CD36 expression alteration. (A) Analysis of CD36 protein levels in different breast cancer cells. (B) Heatmap analysis of differentially expressed genes from transcriptome sequencing of CD36 knockout/overexpression cells. (C) KEGG enrichment analysis of co-altered genes following CD36 knockout and overexpression. (D) GO Enrichment Analysis of Proteomics Results in CD36 Knockout and Overexpressing Cells.

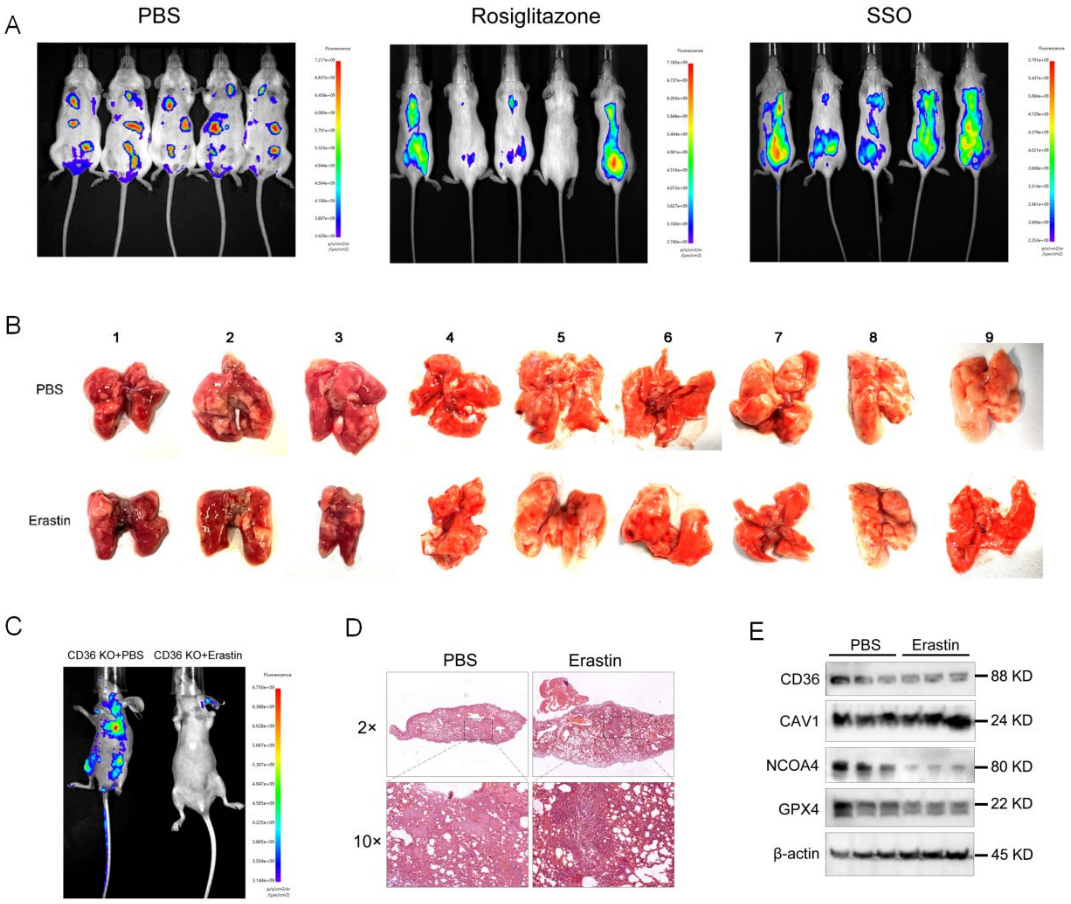

Figure S3: In vivo animal experiments. (A) Fluorescence in vivo imaging to observe the overall metastasis of xenograft tumors in nude mice upon rosiglitazone or SSO treatment. (B) Macroscopic observation of lung tissue sections in nude mice. (C) Using fluorescence in vivo imaging to observe the effect of the ferroptosis activator (Erastin) on the overall metastasis of xenograft tumors in nude mice. (D) H&E staining of lung tissue sections in nude mice. (E) Using Western blot to detect the effect of ferroptosis activator (Erastin) on the protein levels of CD36, CAV1, GPX4, and NCOA4 in nude mouse metastases.
